# The Effect of Sulfated Zirconia and Zirconium Phosphate Nanocomposite Membranes on Fuel-Cell Efficiency

**DOI:** 10.3390/polym14020263

**Published:** 2022-01-10

**Authors:** Rudzani Sigwadi, Touhami Mokrani, Phumlani Msomi, Fulufhelo Nemavhola

**Affiliations:** 1 Department of Chemical Engineering, School of Engineering, University of South Africa, Private Bag X6, Florida 1710, South Africa; tmokrani@unisa.ac.za; 2 Department of Applied Chemistry, University of Johannesburg, Johannesburg 2092, South Africa; pmsomi@uj.ac.za; 3 Department of Mechanical Engineering, School of Engineering, University of South Africa, Private Bag X6, Florida 1710, South Africa; masitfj@unisa.ac.za

**Keywords:** sulphated zirconium oxide, zirconium phosphates, incorporation, water contact angle, fuel-cell efficiency

## Abstract

To investigate the effect of acidic nanoparticles on proton conductivity, permeability, and fuel-cell performance, a commercial Nafion^®^ 117 membrane was impregnated with zirconium phosphates (ZrP) and sulfated zirconium (S-ZrO_2_) nanoparticles. As they are more stable than other solid superacids, sulfated metal oxides have been the subject of intensive research. Meanwhile, hydrophilic, proton-conducting inorganic acids such as zirconium phosphate (ZrP) have been used to modify the Nafion^®^ membrane due to their hydrophilic nature, proton-conducting material, very low toxicity, low cost, and stability in a hydrogen/oxygen atmosphere. A tensile test, water uptake, methanol crossover, Fourier-transform infrared spectroscopy (FTIR), X-ray diffraction (XRD), thermal gravimetric analysis (TGA), and scanning electron microscopy (SEM) were used to assess the capacity of nanocomposite membranes to function in a fuel cell. The modified Nafion^®^ membrane had a higher water uptake and a lower water content angle than the commercial Nafion^®^ 117 membrane, indicating that it has a greater impact on conductivity. Under strain rates of 40, 30, and 20 mm/min, the nanocomposite membranes demonstrated more stable thermal deterioration and higher mechanical strength, which offers tremendous promise for fuel-cell applications. When compared to 0.113 S/cm and 0.013 S/cm, respectively, of commercial Nafion^®^ 117 and Nafion^®^ ZrP membranes, the modified Nafion^®^ membrane with ammonia sulphate acid had the highest proton conductivity of 7.891 S/cm. When tested using a direct single-cell methanol fuel cell, it also had the highest power density of 183 mW cm^−2^ which is better than commercial Nafion^®^ 117 and Nafion^®^ ZrP membranes.

## 1. Introduction

Because of their outstanding conversion efficiency, high power density, and zero pollution emissions, proton-exchange-membrane fuel cells (PEMFCs) are regarded as environmentally acceptable energy-conversion devices for both stationary and portable power applications [1]. Proton-exchange membranes (PEMs) are a key component of PEMFCs because they carry protons between the anode and the cathode while isolating electrons and avoiding fuel crossover. Electrochemical devices that are both durable and efficient, such as PEMFCs and beyond-Li-ion batteries such as Li–sulfur [2,3] and Li–O_2_ batteries [4,5,6]. PEMs such as Nafion^®^117 maintain a greater conductivity and mechanical and chemical stability at lower temperatures in fuel cells [7,8,9,10,11]. The phase of separation between Nafion^®^’s two major monomers (the hydrophobic Teflon-like backbone and the hydrophilic sulfonic-acid-terminated side chain) determines its characteristics. The thermo-chemical environment and material interfaces of Nafion^®^ play a major role in this segregation. However, when run at higher temperatures, these perfluorosulfonic acid membranes face issues such as increased fuel crossover and reduced proton conductivity due to water loss, as well as a higher cost, limiting their use in PEMFCs [12,13]. The insertion of nanosized inorganic fillers into the polymeric matrix to construct hybrid composite membranes has received a lot of interest among those investigating methods to synthesise efficient PEM materials [14]. At low to medium temperatures, the introduction of hygroscopic inorganic nanomaterials such as silica, titanium dioxide, zirconium dioxide, and nanoclays into the polymer matrix has improved features of composite membranes such as water retention capacity and ionic conductivity [15]. These hydrophilic fillers can provide many hydrogen bonding sites, allowing membranes to absorb a large amount of water. When the amount of filler is increased, it weakens the link between the organic polymer and inorganic filler which causes poor interfacial interaction, resulting in a loss of conductivity [6].

When inorganic acid such as sulfated zirconia is calcined at 300 °C, it improves proton conductivity (14.5 mS/cm), with better ion-exchange capacities (IEC) of 0.54 meq/g and greater water uptake due to sulfate ions, which raises the sulfonic acid content inside the membrane [16]. Furthermore, the addition of sulfated zirconia to the membrane gives an additional proton ion within the Nafion matrix. In addition, the modified Nafion^®^ membrane containing S-ZrO_2_ nanoparticles exhibits less swelling, better mechanical properties, and lower methanol permeability. Although mesoporous sulfated zirconia offers the potential to broaden the applications of zirconia-based acid materials, its low thermal stability remains a major drawback, causing the mesoporous sulfated zirconia to collapse when the template is removed at a high temperature. Zirconia is predominantly cationic rather than polyxo in high acidic conditions. However, the polyoxo ions can occur when zirconia is sulfated with ammonia sulfate [Zr (OH)_2_(SO_4_^2−^)_x_(H_2_O)_y_]_n_^n(2−2x)^ [17]. At temperatures above 100 °C, the hydroxyl groups on the oxide surface can effectively retain water molecules and prevent membrane dehydration. Furthermore, incorporating S-ZrO_2_ nanoparticles into Nafion^®^ membranes enhances the sensitivity to high-temperature response. Zirconia oxide is the sole metal oxide with four chemical properties: acidity or basicity and a reducing or oxidizing agent [18].

The fascinating zirconium phosphate (ZrP), a layered acidic inorganic cation-exchange material with the formula Zr(HPO_4_)_2_ 2H_2_O, has been extensively explored [19]. ZrP is known for its great thermal and chemical stability, as well as its high ion conductivity and mechanical strength. Its layered construction enables the incorporation of numerous guest species of diverse sizes between their layers [20,21]. ZrP has been integrated into several polymer-based nanocomposites in recent investigations. These have shown good mechanical, thermal, and barrier properties [22]. The major goal of this article is to use sulfated and phosphated zirconia nanoparticles to modify Nafion^®^ membranes to achieve high proton conductivity, good thermal and chemical stability, and improved water absorption.

## 2. Materials and Methods

### 2.1. Materials

Materials used were phosphoric acid (Sigma), ammonium sulphate (Sigma), zirconium oxychloride hydrate (Merck), sulfuric acid (Merck), Nafion^®^ 117 membrane (Sigma), methanol (Sigma), and hydrogen peroxide (Merck). All of the chemicals were used exactly as they were received, with no further purification.

### 2.2. Membrane Nanocomposite Synthesis

To eliminate contaminants, Nafion^®^ 117 membranes were boiled for 1 h in hydrogen peroxide (3 percent solution), then boiled in sulfuric acid (0.5 M), and finally soaked in distilled water at 80 °C for 1 h [23,24]. After pre-soaking the pure membranes in methanol to open the pores, 5 wt% of ZrP [9] and 5 wt% of S-ZrO_2_ [25] nanoparticles were added. The membranes were soaked five times before being heated at 100 °C for 2 h [26]. The obtained solution was maintained at room temperature for 24 h. The thicknesses of the membranes were measured using a digital micrometer (0.18 mm). To record the accurate number, the thickness reading was recorded more than thrice.

### 2.3. Characterisations

Fourier-transform infrared spectroscopy (FTIR), scanning electron microscopy (SEM), X-ray diffraction (XRD) examination, and thermal gravimetric analysis (TGA) were used to characterize the membranes.

### 2.4. Tensile Test

Under a uniaxial testing system, the mechanical strength of membranes was measured. The breadth, thickness, and length were all measured using a Vernier calliper. All membranes had a clamping area of 4 mm × 10 mm. The tension applied to the sample was calculated using the observed thickness of 0.18 mm. Membranes were measured at 25 °C using the CellScale Ustretch instrument with actuator speeds of 40, 30, and 20 mm/min.

### 2.5. Measurements of the Water Contact Angle

Contact angles were used to determine the hydrophilicity of the membrane surfaces (Phoenix 300 contact angle analyser instrument equipped with a video system). For analysis, the membrane was cut into strips and put on glass slides. By putting the tip of the syringe close to the sample surface, a droplet of deionized water (0.16 L) was placed onto the surface of membranes at ambient temperature. To get an average value, the measurement was performed ten times at various membrane surfaces. The wetting process was recorded prior to the water droplet adhering to the sample’s surface until there was no more noticeable change at the surface.

### 2.6. Water Uptake (WU) and Swelling Ratio (SR)

The membranes were immersed in distilled water for 24 h at different temperatures of 80 °C, 60 °C, and 30 °C and then weighed and measured. Using the equations below, the water uptake and swelling ratio of soaked membrane were calculated:

(1) $$W_{\mathrm{up}}=\frac{\left(m_{\mathrm{wet}}-m_{\mathrm{dry}}\right)}{m_{\mathrm{dry}}}\times 100\%$$

(2) $$\mathrm{SR}=\frac{\left(L_{w}-L_{d}\right)}{L_{d}}\times 100\%$$

where W_up_ is the WU percentage, m_wet_ the membrane wet mass, m_dry_ the membrane dry mass, L_w_ the membrane wet length, and L_d_ the dried length of the membrane.

### 2.7. Ion-Exchange Capacity (IEC)

The IEC of membranes was determined using the equation below based on the titrated results:

(3) $$\mathrm{IEC}=\frac{V_{\mathrm{NaOH}\;}{\times \;C}_{\mathrm{NaOH}}}{m_{d}}$$

where V_NaOH_ is the volume of titrated NaOH, C_NaOH_ the concentration of NaOH and the membrane dried mass is m_d_.

### 2.8. Measurements of the Methanol Permeability

A two-compartment permeation-measuring cell was used to determine the methanol crossover. Methanol solution (50 mL) was placed in compartment (A) and distilled water in compartment (B) (50 mL). With a diffusion area diameter of 3.5 cm, the membrane was installed between the two compartments. The readings were collected at 30 °C, 60 °C, and 80 °C using 5 M and 2 M methanol solutions. The following equation was used to compute methanol permeability (P):

(4) $$C_{B}=\frac{A\;P}{V_{B}\;L}{\;C}_{A}\;\left(t-t_{o}\right)$$

where C_B_(t) is the methanol concentration in compartment B at time t; methanol content in compartment A is denoted by C_A_ and in compartment B, V_B_ represents the volume of distilled water, the effective permeating area is A, and the membrane thickness is L.

### 2.9. Measurement of the Proton Conductivity

A four-point probe conductivity cell was used to measure the conductivities of the membranes. At 0.1 mA current and 1 MHz to 10 Hz frequency, the proton conductivity was measured galvanostatically and estimated using the equation below:

(5) $$\sigma =\frac{L}{{\mathrm{AR}}_{s}\;}$$

where R_s_ denotes the measured membrane resistance, the area of the membrane normal to the current flow is A, and the thickness of the membrane is L.

### 2.10. The Cell Polarization and the Fabrication of Membrane Electrode Assembly

The performance of the membranes was tested using a direct methanol fuel cell (DMFC). The MEA was prepared by using 20% Pt Vulcan XC-72R in Nafion^®^ solutions for ink preparation and Pt in carbon cloth. Pt on carbon cloth was used for the anode and cathode membrane electrode assemblies (MEA). The MEA was put together without the use of hot pressing. At 60 °C, fuel cells were tested with a 2 M methanol solution. On a single fuel-cell test, the galvanostatic potential of the fuel cell was measured in the open air.

## 3. Results and Discussion

### 3.1. Fourier-Transform Infrared

**Figure 1:**
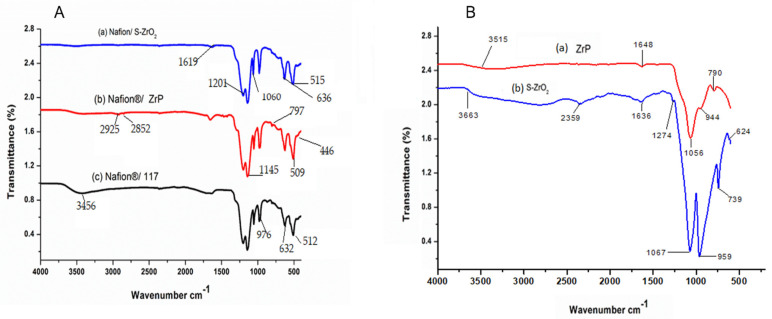
FTIR spectra of (**A**): (a) Nafion^®^ S-ZrO_2_ and (b) Nafion^®^ ZrP nanocomposite membranes and (c) Nafion^®^ 117 membrane and FTIR spectra of (**B**): (a) S-ZrO_2_ and (b) ZrP nanoparticles.

Figure 1A shows the FTIR spectra of Nafion^®^ S-ZrO_2_ and Nafion^®^ ZrP nanocomposite membranes in comparison to Nafion^®^ 117 membrane and Figure 1B shows the FTIR spectra of S-ZrO_2_ and ZrP nanoparticles. Figure 1A(a–c) shows that the O-H stretching vibration of the membranes is 3456 cm^−1^, which corresponds to physically adsorbed water [27,28]. However, as shown in Figure 1A(a–c), the peaks at 3456 cm^−1^ for Nafion^®^ S-ZrO_2_ and Nafion^®^ ZrP nanocomposite membranes are significantly lower than those for commercial membranes. This may be due to the incorporation of nanoparticles into the nano-composite membranes, which increases the water content. Figure 1A(a–c) shows the O-H bending vibration of free water molecules at 1630 cm^−1^, due to symmetric S-O stretching, the membranes have a comparable peak at 1060 cm^−1^ [29,30] and a band at 1145 cm^−1^ and 1201 cm^−1^ were formed due to symmetric C-F stretching [31]. Furthermore, the C-O-C stretching caused the peaks at 976 cm^−1^ and the 512 cm^−1^ band was due to symmetric O-S-O bending, whereas the 632 cm^−1^ band was due to C-S group stretching [31,32]. Asymmetric stretching vibrations of the Zr-O-Zr bond were also assigned to the peaks at 636 cm^−1^ and 515 cm^−1^, respectively, which were identical to the Nafion^®^ 117 membrane’s transmittance peaks [33]. This could be due to the Nafion^®^ matrix’s well-distributed inorganic components. The bands at 1619 cm^−1^ were allocated to H-O-H bending vibration mode in Figure 1A(a), which was slightly similar to the bands at 1648 cm^−1^ and 1636 cm^−1^ for ZrP and S-ZrO_2_ as shown Figure 1B(a,b); this may have been due to the sulfate group’s coordinated molecular water [33]. The peaks of Zr-O and P-O_4_ can be seen in Figure 1A(b) at 797 cm^−1^, 509 cm^−1^, and 446 cm^−1^, respectively, this could be due to ZrP nanoparticles embedded in the Nafion^®^ membrane and the C–H stretching of the modified Nafion^®^ membrane, with stretch vibrations between 2925 cm^−1^ and 2852 cm^−1^ [34,35].

### 3.2. Membrane Morphology

**Figure 2:**
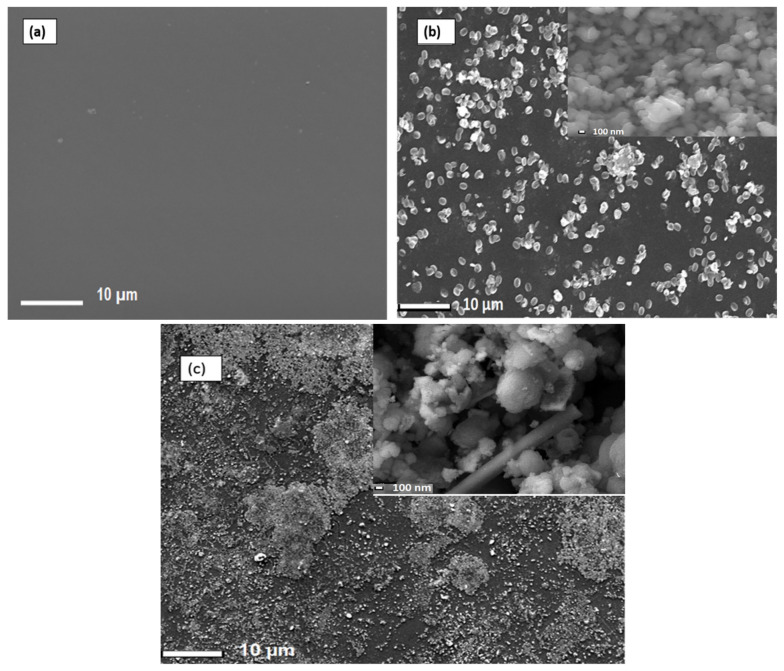
SEM micrograph of (**a**) Nafion^®^ 117 membrane and (**b**) Nafion^®^ ZrP (insert: ZrP nanoparticles) and (**c**) Nafion^®^/S-ZrO_2_ nanocomposite membranes (insert:S-ZrO_2_ nanoparticles).

**Figure 3:**
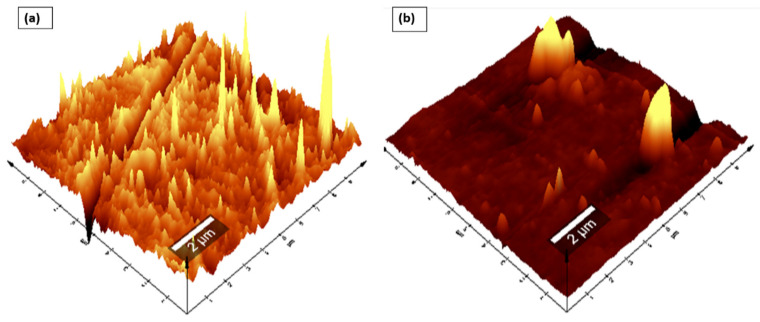
AFM (**a**,**b**) topography amplitude image of Nafion^®^/S-ZrO_2_ and Nafion^®^/ZrP nanocomposite membranes.

To produce Nafion^®^/ZrP and Nafion^®^/S-ZrO_2_ nanocomposite membranes, 5 wt% nanoparticles (ZrP and S-ZrO_2_) were incorporated into commercial Nafion^®^ 117 membrane. The morphology of the obtained membranes was examined using scanning electron microscopy (SEM). Figure 2a shows that the Nafion^®^ 117 membrane is dark in colour and free of nanoparticles. Figure 2b shows a Nafion^®^/ZrP nanocomposite membrane with uniformly distributed ZrP nanoparticles and fewer agglomerates in the membrane matrix. This can also be seen in SEM micrographs of ZrP nanoparticles, which show the presence of well-oriented nanoparticles with a very smooth surface, as shown in the Figure 2b insert. Figure 2c shows the significant difference in surface morphologies observed under modified Nafion^®^ 117 with S-ZrO_2_ nanoparticles that were well scattered and agglomerated. As illustrated in the Figure 2c insert, this could be because the synthesized sulfated zirconia was made into the tiniest particles, which clustered together and agglomerated in their varied shapes. The electrodes are expected to have the highest proton conductivity (ionic conductive groups of sulfated zirconia exist on its solid surface) as confirmed in Table 1 [36]. Figure 3 shows three-dimensional atomic force microscopy (AFM) surface images for Nafion^®^/S-ZrO_2_ and Nafion^®^/ZrP nanocomposite membranes at a scan size of 10 µm by 10 µm. Figure 3a,b shows that the surface roughness of Nafion^®^/S-ZrO_2_ and Nafion^®^/ZrP nanocomposite membranes was 41.46 nm and 18.59 nm, respectively, on topography images. The rougher surface of modified Nafion^®^ nanocomposite membranes increases electrode contact [37]. The brightest areas in these images show the highest point of the membrane surface, while the dark areas show the valleys or membrane holes, as seen in Figure 3a,b. The surface roughness of the Nafion^®^/S-ZrO_2_ membrane was higher than that of the Nafion^®/^ZrP nanocomposite membranes, as numerous small peaks and valleys were replaced by many small ones, resulting in a smooth membrane surface (Figure 3a) and Table 1 [38]. Figure 3b shows the dark spots which are made up of a polymer matrix that does not contain any nanoparticles [39]. Furthermore, Figure 3a shows the inadequate bright spots which indicate the appropriate distribution and aggregation of particles in a Nafion^®^ matrix [39].

### 3.3. Analysis of XRD Structure

**Figure 4:**
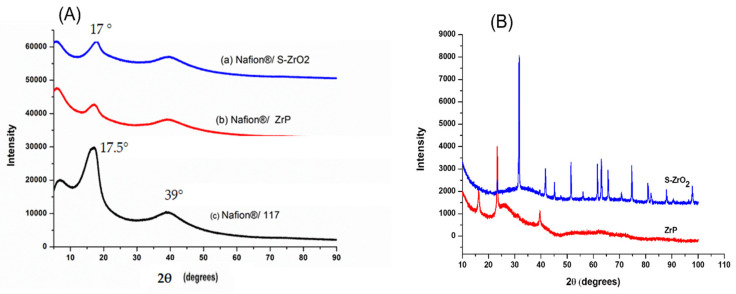
(**A**) XRD patterns of (a) Nafion^®^/S-ZrO_2_ and (b) Nafion^®^/ZrP nanocomposite membranes and (c) Nafion^®^ 117 membrane, (**B**) XRD patterns of S-ZrO_2_ and ZrP nanoparticles.

Figure 4A illustrates the XRD diffraction patterns of Nafion^®^/S-ZrO_2_ nanocomposite membranes, commercial Nafion^®^ 117 membranes, and Nafion^®^/ZrP nanocomposite membranes, respectively. Figure 4A(a,b) reveals that the diffraction peaks of the Nafion^®^/S-ZrO_2_ and Nafion^®^/ZrP nanocomposite membranes are at 17°, which is slightly lower than that of the commercial membrane [40]. These can also be seen on the modified membranes’ diffraction peaks at 39° in Figure 4A(a,b), which are slightly lower than the commercial membrane. This could be due to the well-distributed nanoparticles within the Nafion^®^ matrix, as confirmed by SEM results, which reduces the intensity of the diffraction peak. The powder XRD patterns of the produced S-ZrO_2_ and ZrP nanoparticles are shown in Figure 4B. The structure of ZrP is shown by a series of distinctive reflections in the range of 0–50°, whereas the distinctive reflections of S-ZrO_2_ are in the 0–100° range. Figure 4A(c) indicates that the commercial Nafion^®^ 117 membrane only has two diffraction peaks at 17.5° and 39° 2θ, this is due to the ionomer’s perfluorocarbon chains being semi-crystalline [41]. As a result of the broken hydrogen bonding within the Nafion^®^ 117 membrane, membranes incorporating nanoparticles tend to be amorphous with a decrease in crystallinity.

### 3.4. Thermo-Gravimetric Analysis (TGA)

**Figure 5:**
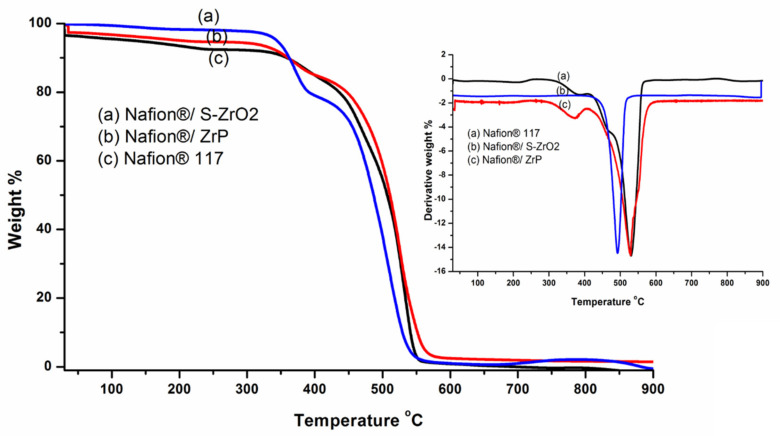
The TGA and DTG of (**a**) Nafion^®^/S-ZrO_2_ and (**b**) Nafion^®^/ZrP nanocomposite membranes and (**c**) Nafion^®^ 117 membrane.

TGA was used to determine the derivative thermogravimetric (DTG) and thermal stability of modified membranes and Nafion^®^ 117 membranes. To assess the thermal properties of the membranes, thermal stability tests were carried out. Thermal stability is critical in defining the operating temperature of a fuel-cell application. The TGA results of the Nafion^®^ 117 membrane, Nafion^®^/ZrP, and Nafion^®^/S-ZrO_2_ nanocomposite membranes follow a three-stage deterioration pattern, as shown in Figure 5. The first step corresponded to absorbed water evaporation, thermal degradation’s second stage, the polymer matrix was then thermally oxidized in the third stage. The thermal stability of modified Nafion^®^ membranes with S-ZrO_2_ nanoparticles was better than that of modified Nafion^®^ membranes with ZrP nanoparticles in Figure 5(a,b), as it began to lose weight at temperatures above 300 °C, whereas Nafion^®^/ZrP began to lose weight at temperatures below 150 °C. This could have been due to the well-distributed S-ZrO_2_ in the form of small particles, as SEM results show. Furthermore, at around 150 °C, Nafion^®^/ZrP began to lose weight, which corresponded to water adsorption as shown in Figure 5(b). The decomposition of the sulfonic acid groups caused the second weight loss at 340 °C [42]. The degradation of the polymer backbone chain may have been the cause of the third weight loss at 570 °C. This decreased thermal degradation could be attributed to the inorganic filler’s composition and intimate interaction with the hydrophobic Nafion^®^ backbone, as opposed to the commercial Nafion^®^ 117, which decomposed at 380 °C [43]. Figure 5 (DTG insert) shows that the nanocomposite membranes had better heat stability about 340 °C, but the Nafion^®^ 117 membrane had better thermal stability up to 240 °C (DTG insert). This could be because of the inorganic nanofillers used in Nafion^®^ membranes [44] that operate as a better insulator and mass transport barrier to the volatile compounds produced during decomposition. As a result, it is ideal for fuel-cell applications. Due to the evaporation of adsorption bound water to the sulfonic groups, the commercial Nafion^®^ 117 membrane in Figure 5(c) initially lost weight at 100 °C. [8]. At 380 °C, the second weight loss could be attributed to sulfonic group degradation [42]. The degradation of the polymer backbone chain may have been the cause of the third weight loss at 550 °C [45]. We may conclude that reducing the mobility of the Nafion^®^ chain delays the initial weight loss and thermal degradation of modified membranes compared to unmodified membranes.

### 3.5. Tensile Tests

**Figure 6:**
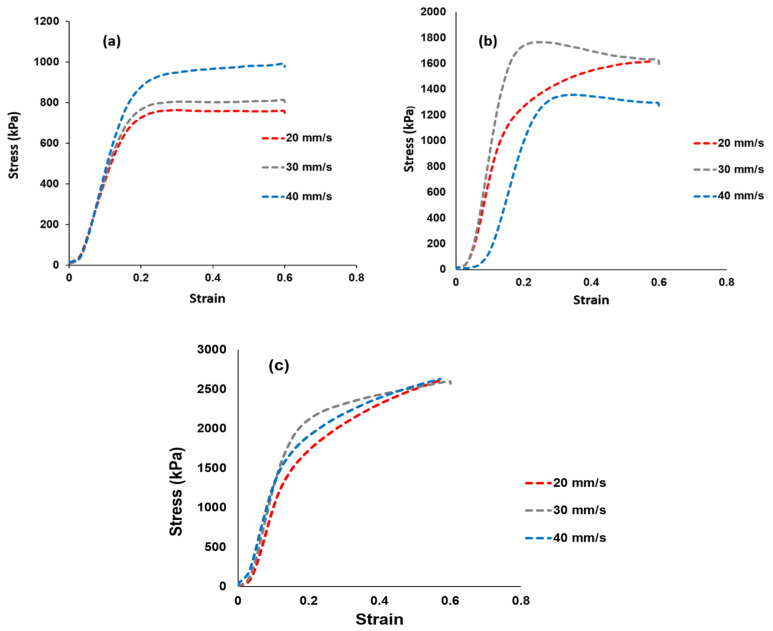
Mechanical tensile tests results of (**a**) Nafion^®^ 117 membrane and (**b**) Nafion^®^/ZrP and (**c**) Nafion^®^/*S*-ZrO_2_ nanocomposite membranes shows stress versus strain ratio curve.

Tensile tests were used to determine the membrane’s mechanical strength and the findings are shown in Figure 6. Figure 6a–c shows the stress–strain curves of the Nafion^®^ 117 membrane and the Nafion^®^/ZrP and Nafion^®^/S-ZrO_2_ nanocomposite membranes at 20, 30, and 40 mm/min [46,47,48]. The elasticity and flexibility of the membranes at 0.6 stress versus strain are demonstrated at a stress rate of 20 mm/min. The modified membrane with inorganic nanofiller improved the tensile strength within the membrane, as shown in Figure 6b,c, which could be attributed to the nanofiller’s incorporation into the Nafion^®^ matrix. When ZrP was added to Nafion^®^, the tensile stress was lowered to 1300 kPa at a strain rate of 40 mm/min, this could have been due to the small agglomeration of ZrP nanoparticles in the Nafion^®^ matrices, which resulted in the modified membrane being brittlely fractured, whereas the Nafion^®^/S-ZrO_2_ shows a greater tensile stress of 2630 kPa at the same strain rate. This could be attributed to well-distributed S-ZrO_2_ with minimal agglomeration, as seen by SEM and AFM data, as aggregated nanoparticles may have had an impact on mechanical strength. Furthermore, good contact between the membrane and nanoparticles would improve nanocomposite reinforcement and fuel-cell durability, which is a more important requirement for the production and operating process. Figure 6a–c shows the Nafion^®^/S-ZrO_2_ tensile stress–strain curves, which demonstrate a significant improvement as it achieved a tensile stress of 2630 kPa at 20, 30, and 40 mm/min, which was twice that of Nafion^®^/ZrP (1630 kPa) and Nafion^®^ 117 (990 kPa). The enhanced tensile stress of Nafion^®^/S-ZrO_2_ membranes may be related to the presence of ammonia sulphate ions within the membrane, which promote the movement and flexibility of polymer chains, resulting in mechanical strength suitable for fuel-cell applications. Furthermore, the nanocomposite membrane had a higher stress–strain than the Nafion^®^ 117 membrane. Overall, the results demonstrated that adding sulfated zirconia to the Nafion^®^ membrane improved the stress–strain properties, which are a good DMFC features [49].

### 3.6. Methanol Permeability

**Figure 7:**
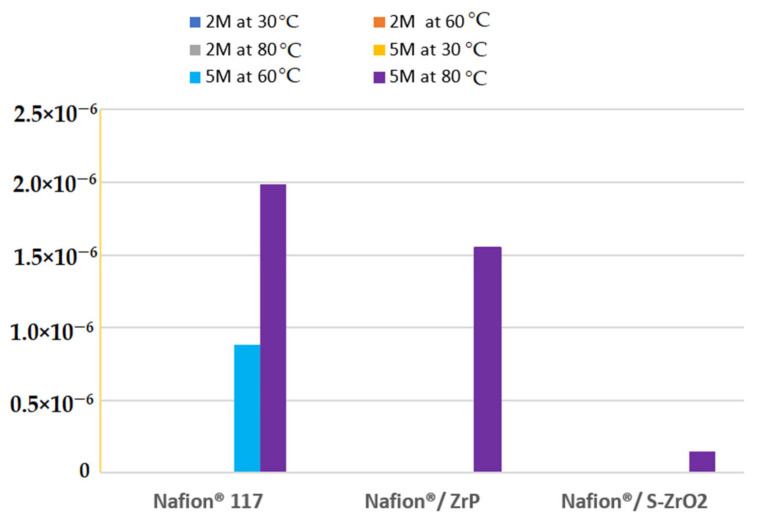
The methanol permeability of Nafion^®^ 117 membrane and Nafion^®^/ZrP and Nafion^®^/ZrO_2_ nanocomposite membranes at 5 M and 2 M concentration.

At different methanol concentrations (2 M and 5 M) and temperatures of 30 °C, 60 °C, and 80 °C, the methanol permeability of Nafion^®^ 117 membrane and Nafion^®^/ZrP and Nafion^®^/S-ZrO_2_ nanocomposite membranes was measured. There was no methanol crossover seen for all membranes at varied temperatures and lower concentrations of 2 M methanol [50] as shown in Figure 7. Figure 7 shows that a membrane’s methanol crossover is influenced by its affinity for both water and methanol, as well as the amount of empty space within the membrane [51]. However, because of the nanocomposite’s dense internal structure and greater filler loading, the methanol molecules have a longer diffusion path. As a result, the permeability of methanol in nanocomposite membranes decreases. Furthermore, because methanol permeability is caused by the movement of molecules across the membrane, the size of the transport molecules must be considered while analysing methanol permeability. According to Yang et al., lowering the methanol concentration lowers the methanol crossover because the concentration gradient is lower [52]. As a result, a higher concentration of 5 M methanol solution was used in this study. At 60 °C, the methanol permeability of Nafion^®^ 117 membrane and Nafion^®^/ZrP and Nafion^®^/S-ZrO_2_ nanocomposite membranes was 8.84 × 10^−7^ cm^2^/s, 0 cm^2^/s, and 0 cm^2^/s (no crossover), respectively, as shown in Figure 7. The methanol permeability of modified and unmodified Nafion^®^ membranes increased as the temperature rose, as shown in Figure 6. When the temperature is raised to 80 °C, the results demonstrate that nanocomposite membranes have a lower methanol penetration, indicating that water permeation is greater than methanol permeation at high temperatures. This is because methanol molecules are larger than water molecules and are more likely to be obstructed by space limits inside the membrane structure [51]. As shown in Figure 7, the methanol permeability of Nafion^®^ 117 membrane and Nafion^®^/ZrP and Nafion^®^/S-ZrO_2_ nanocomposite membranes was 1.99 × 10^−6^ cm^2^/s, 1.55 × 10^−6^ cm^2^/s, and 1.50 × 10^−7^ cm^2^/s, respectively. The nanocomposite membrane had a lower methanol permeability than commercial Nafion^®^ 117, which was due to the addition of ZrP and S-ZrO_2_ to Nafion^®^ 117, which improved the barrier properties of Nafion^®^ membrane towards methanol. Furthermore, by preventing methanol from migrating through the membrane, the well-dispersed nanoparticles may limit methanol crossing [53]. Because methanol crossover can affect fuel efficiency, a reduced or low methanol crossover is critical in DMFC applications. In addition, modified Nafion^®^ nanocomposite membranes appear to be potential electrolytes for use in fuel cells.

### 3.7. Water Contact Angle, Water Uptake, Dimensional Swelling Ratio, Ion-Exchange Capacity, and Proton Conductivity Measurement

**Figure 8:**
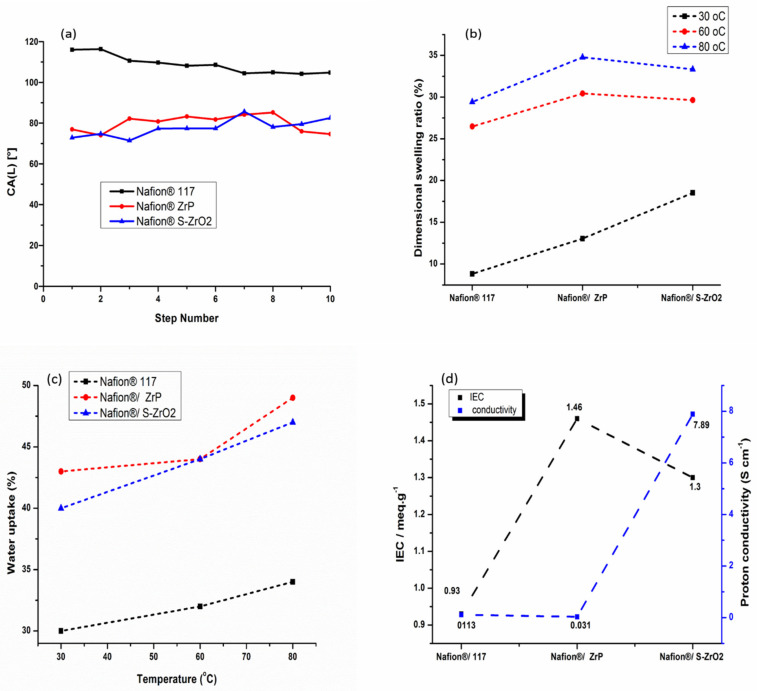
Contact angle (**a**), linear expansion (**b**), water uptake (**c**), and ion-exchange capacity and proton conductivity measurement (**d**) of Nafion^®^ 117 membrane, Nafion^®^/ZrP and Nafion^®^/S-ZrO_2_ nanocomposite membranes.

In fuel-cell applications, water wettability within the membrane matrix is critical because it promotes protonic conductivity of the membrane by allowing protons to move through it [40]. Figure 8a shows how contact angle was used to determine water wettability. A polymer with a smaller contact angle is more hydrophilic, while high contact angle indicates a more hydrophobic polymer. Because of its hydrophobic nature, the commercial Nafion^®^ 117 membrane attained a contact angle larger than 90°, as illustrated in Figure 8a [10]. As shown in Figure 8a, the contact angle of Nafion^®^/S-ZrO_2_ and Nafion^®^/ZrP nanocomposite membranes was smaller, ranging from 80° to 68°, this could be owing to the introduction of inorganic material with a hydrophilic property that holds water [54]. In addition, the modified membranes demonstrated that inorganic material impregnating the Nafion^®^ membrane surface results in hydrophilicity [55]. The hydrophobicity of Nafion^®^ membranes increased when they are treated with hydrophobic nanoparticles. The dimensional swelling ratio at 30 °C, 60 °C, and 80 °C showed a slightly increase with the increases in temperature as shown in Figure 8b and Table 2. However, when the Nafion^®^/ZrP nanocomposite membrane was soaked at the higher temperature of 80 °C, a higher dimensional swelling ratio of 35% was obtained when compared with Nafion^®^ 117 membrane (29%) and Nafion^®^/S-ZrO_2_ nanocomposite membrane (33%). Moreover, when the temperature increased, it also increased the dimensional stability and water uptake of the membranes.

Figure 8c and Table 2 shows the water uptake of Nafion^®^ 117 membranes and Nafion^®^/ZrP nanocomposite membranes, and Nafion^®^/S-ZrO_2_ nanocomposite membranes at 30 °C, 60 °C, and 80 °C. As the temperature rose from 30 °C to 80 °C, all membranes exhibited an increase in water uptake [56]. At 80 °C, the Nafion^®^/ZrP and Nafion^®^/S-ZrO_2_ nanocomposite membranes had the highest water uptake of 49% and 47%, compared to 34% for Nafion^®^ 117 membranes as shown in Figure 8(c). This could be due to the use of hydrophilicity of the ZrP nanoparticles, which helped the membranes retain water [57,58]. Moreover, this could be attributed to an excellent distribution of hygroscopic S-ZrO_2_ nanoparticles that hold water within the membrane matrix. Table 1 shows that the modified membrane with ZrP and S-ZrO_2_ nanoparticles demonstrated enhanced water uptake at a higher temperature of 60 °C than the unmodified membrane. This could be attributed to the hydrophilic character of acidic nanoparticles, which raises the acidity and surface areas of nanoparticles integrated into the Nafion^®^ matrix, as well as the existence of a high concentration of polymer-filler interfaces, which increases the free volume [59]. Furthermore, nanoparticle impregnation causes clusters in the pore of the Nafion^®^ membrane, resulting in the nanocomposite membrane’s higher water uptake [40,60,61].This conclusion is consistent with the hydrophobic site’s reduced contact angle in Figure 8a.

The proton conductivity and IEC of Nafion^®^ 117 membranes, Nafion^®^/ZrP nanocomposite membranes, and Nafion^®^/S-ZrO_2_ nanocomposite membranes are shown in Figure 8d and Table 2. The Nafion^®^/ZrP and Nafion^®^/S-ZrO_2_ nanocomposite membranes had an IEC of 1.46 meg/g and 1.3 meg/g greater than the Nafion^®^ 117 membrane’s IEC of 0.93 meg/g. This could be because acidic nanoparticles are impregnated into the Nafion^®^ membrane, which provides the membrane with a strong acid site [58], with the inclusion of sulfate ions as proton-exchange sites within the Nafion^®^ matrix [62]. The nanocomposites’ IEC rises as more nanoparticles are incorporated into the membrane. The proton conductivity of a polymer electrolyte membrane in a fuel cell is the most essential factor that influences its performance. At room temperature, the proton conductivity of the Nafion^®^/ZrP nanocomposite membrane was 0.031 Scm^−1^, compared to 7.89 Scm^−1^ and 0.113 Scm^−1^ for the Nafion^®^/S-ZrO_2_ nanocomposite membrane and Nafion^®^ 117 membrane. It is possible that zirconia phosphate nanoparticles within the membranes are causing this decrease in proton conductivity [11,63] because their ionic activity and water mobility are both affected by high temperatures. Furthermore, as the length of the hydrophilic block rises, so does their ionic conductivity. In addition, sulphating zirconia nanoparticles with NH_3_SO_4_ acid improved the proton conductivity of the nanocomposite membranes by promoting the migration of sulfonated groups to form cluster aggregates via the strong electrostatic contacts of the Na^+^ counter ions.

### 3.8. Fuel-Cell Performance

**Figure 9:**
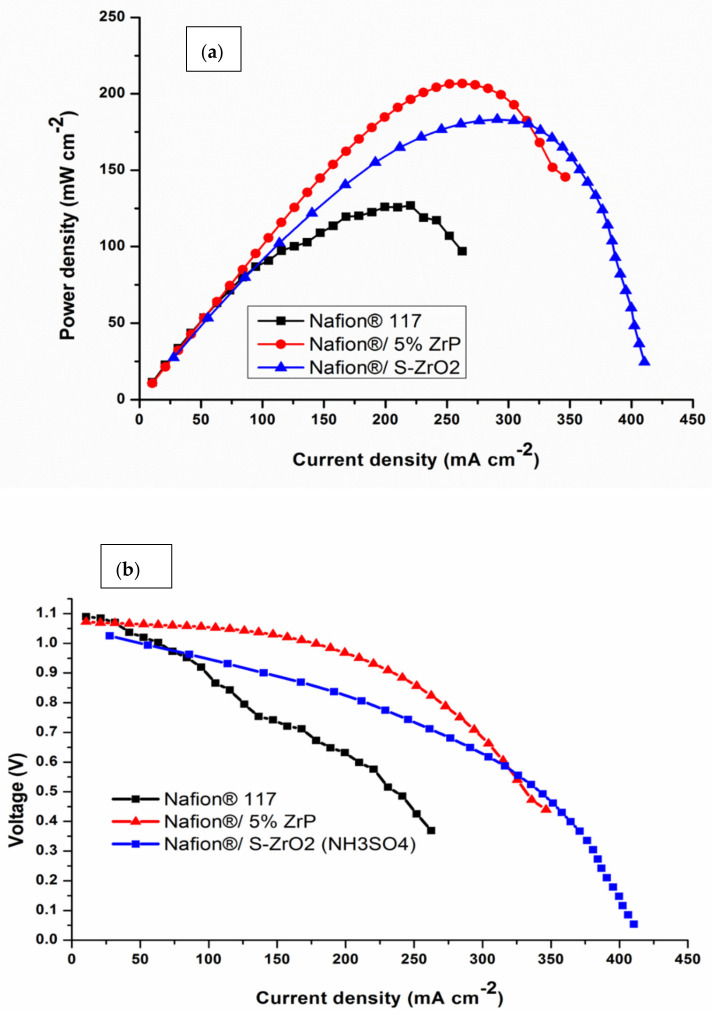
(**a**,**b**) DMFC polarization of Nafion^®^ 117 membrane and Nafion^®^/ZrP and Nafion^®^/S-ZrO_2_ nanocomposite membranes obtained at 60 °C.

Single-cell DMFC tests were done at 60 °C to further confirm the influence of acidic nanoparticles on the electrochemical performance of commercial Nafion^®^ 117 membrane. The polarization and power density graphs for DMFCs are shown in Figure 9 and Table 3. The peak density of the Nafion^®^/ZrP nanocomposite membrane was 206.79 mW cm^−2^, which is greater than the Nafion^®^/S-ZrO_2_ nanocomposite membrane (183 mW cm^−2^) and Nafion^®^ 117 membrane (126.04 mW cm^−2^) at the current densities of 189 mA cm^−2^. Therefore, Nafion^®^ 117 membrane incorporated with ZrP obtained higher power density (145 mW cm^−2^) than commercial membrane, with current density of 350 mA cm^−2^ as shown in Figure 9a. This may have been due to the nanoparticles being well deposited within the membrane pores, that are good at water retention and enhance the conductivity of modified membrane [64]. The best fuel-cell performance is ascribed to the better water retention capabilities of the composite membrane with acidic nanoparticle filler. Furthermore, the increased power density could be attributable to the use of ZrP, which reduces the ohmic resistance of the Nafion^®^ membrane [65]. The Nafion^®^/S-ZrO_2_ nanocomposite membrane’s superior performance in DMFC is attributed to its proton conductivity and decreased methanol permeability. The modified membrane had a higher voltage than the commercial membrane, as seen in Figure 9b, when compared to the Nafion^®^ 117 membrane (0.58 V), and Nafion^®^/ZrP (0.91 V) and Nafion^®^/S-ZrO_2_ (0.85 V) nanocomposite membranes at current densities of 200 mAcm^−2^. This indicates that the nanocomposite membranes are a good barrier to prohibit the crossover of both the fuel and the oxidant. Furthermore, this could be attributed to a larger percentage of ZrP in the Nafion matrix membrane. The improvement in voltage and current density can be seen by the decreased weight percent incorporation. This could be due to the nanoparticles that had been well deposited within the membrane pores, which aided in water retention and improved the modified membrane’s conductivity. [64]. The Nafion^®^/ZrP nanocomposite membrane (0.48 V) displays a modest drop in voltage at current densities of 350 mAcm^−2^. Although the Nafion^®^/ZrP membrane outperformed the Nafion^®^/S-ZrO_2_ nanocomposite membrane in terms of fuel-cell performance, the Nafion^®^/S-ZrO_2_ nanocomposite membrane showed long-term stability. Therefore, we can conclude that the Nafion^®^/S-ZrO_2_ nanocomposite membranes are reasonably decent and appropriate for DMFC applications. Also, these results suggest that modified membranes show great potential in direct methanol fuel cells.

## 4. Conclusions

The impregnation approach was used to successfully construct Nafion^®^/ZrP and Nafion^®^/S-ZrO_2_ nanocomposite membranes with low methanol permeability and high proton conductivity. Because of the nature of an inorganic fillers and their tight interaction with the hydrophobic Nafion^®^ backbones, the thermal stability of the nanocomposite membranes began to degrade at a high temperature of 450 °C. Furthermore, when compared to Nafion^®^ 117 membrane, water uptake, IEC, and linear expansion of nanocomposite membranes were improved. The results revealed that the nanocomposite membranes obtained a lower water contact angle than the commercial Nafion^®^ membrane. Moreover, the results show that the incorporating of S-ZrO_2_ in Nafion^®^ membrane enhances the conductivity compared to membrane modified with ZrP nanoparticles. The results demonstrate a decrease in methanol permeability on modified Nafion^®^ membrane, at a higher temperature of 80 °C and a 5 M methanol concentration when compared to Nafion^®^ 117 membrane, which may be due to the incorporation of inorganic components within the membranes. The improved membrane’s lower methanol permeability and strong proton conductivity resistance further verified its feasibility for use in fuel cells. The inclusion of ZrP and S-ZrO_2_ in the membranes was confirmed by SEM and FTIR findings, which also improved water uptake. At 80 °C, the Nafion^®^/ZrP and Nafion^®^/S-ZrO_2_ nanocomposite membranes’ water uptake and swelling ratio ranged from 47 to 49% and 33 to 35%, respectively. These findings suggest that nanocomposite membranes have higher IEC with improved conductivity. The power density of the Nafion^®^/ZrP (206.79 mW cm^−2^) and Nafion^®^/S-ZrO_2_ (183 mW cm^−2^) nanocomposite membranes was higher than that of the commercial Nafion^®^ 117 membranes (126 mW cm^−2^). The Nafion^®^/S-ZrO_2_ nanocomposite membrane produced a maximum power density of 188.6 mWcm2 and an OCV of 0.98 V, indicating that Nafion^®^/S-ZrO_2_ nanocomposite membranes are promising for fuel cells. The results also showed that membrane modified with ZrP nanoparticles obtained the highest fuel-cell performance at maximum power density of 188.6 mW cm^−2^ and an OCV of 0.98 V with the short life when compared with Nafion^®^/S-ZrO_2_ which attained a long-life in the fuel-cell performance.

## Figures and Tables

**Table 1:** Surface roughness parameters of Nafion^®^/S-ZrO_2_ and Nafion^®^/ZrP nanocomposite membranes.

Sample	Roughness		
	*R_q_* (nm)	*R_a_* (nm)	*Peak-Peak* (nm)
Nafion^®^/S-ZrO_2_	41.4604	24.2613	717.253
Nafion^®^/ZrP	18.5894	8.36209	228.592

**Table 2:** The proton conductivity and IEC of Nafion^®^ 117 membrane and Nafion^®^/ZrP and Nafion^®^/S-ZrO_2_ nanocomposite membranes.

Membranes	Nafion^®^ 117	Nafion^®^/ZrP	Nafion^®^/S-ZrO_2_
IEC (meq/g)	0.93	1.46	1.3
Proton conductivity (S/cm) at 25 °C	0.113	0.031	7.89
Water uptake % (30 °C)	30	43	40
Water uptake % (60 °C)	32	44	44
Water uptake % (80 °C)	34	49	47

**Table 3:** A comparison of the power density of the synthesised nanocomposite membranes and commercial Nafion^®^ 117 in this investigation with those of membranes reported on in various research articles.

Membranes	Power Density (mW.cm^−2^)	Operating Temperature (°C)	Reference
Nafion^®^ 117	126.04	60	Current study
Nafion^®^/S-ZrO_2_	206.79	60	Current study
Nafion^®^/ZrP	183	60	Current study
Nafion/ZP2	35.9	60	[66]
Nafion^®^ 117	50.1	60	[67]
ZrP/Nafion115	96.3	75	[68]
FZP-9110	35	60	[68]
Nafion/sb-CD (NC5)	58	25	[69]

## Data Availability

All materials for this study are presented in this article and available on request to the corresponding authors.
